# The Effect of Functionally Graded Materials on Temperature during Frictional Heating at Single Braking

**DOI:** 10.3390/ma14216241

**Published:** 2021-10-20

**Authors:** Aleksander Yevtushenko, Katarzyna Topczewska, Przemysław Zamojski

**Affiliations:** Faculty of Mechanical Engineering, Bialystok University of Technology (BUT), 45C Wiejska Street, 15-351 Bialystok, Poland; a.yevtushenko@pb.edu.pl (A.Y.); zamojski.przemyslaw@gmail.com (P.Z.)

**Keywords:** functionally graded materials, braking, frictional heating, temperature

## Abstract

A mathematical model for evaluation of the temperature mode of the disc–pad system during single braking is proposed. The model is based on the thermal problem of friction formulated for two semi-infinite bodies, compressed with pressure increasing over time while reducing the sliding velocity from the initial value to zero at the stop. The exact solution to this problem was obtained by means of Duhamel’s theorem. Validation of the solution was performed by achieving in special cases parameters of known solution to this problem with constant pressure and velocity (under uniform sliding). The results of the numerical calculations are presented for a selected friction pair, made of functionally graded materials with titanium alloy (disc) and aluminum alloy (pad) cores coated with ceramics graded toward friction surfaces. For the established values of the parameters such as the rise time in pressure and the FGM gradients, the ability to quickly obtain spatiotemporal temperature distributions in the disc and pad was presented. The influence of the variability of these parameters on the maximum temperature of the brake system was also investigated.

## 1. Introduction

Friction elements of braking systems are subjected to severe conditions such as high temperature and intensive wear. During braking, performance of these components in terms of efficiency, service life, and dissipation of heat from the contact surface depends on the operating conditions and material properties. It has been shown that the operating characteristics of the entire assembly of the braking system can be significantly improved by introducing a smooth gradient in the microstructure of the friction materials [1]. Such functionally graded materials (FGMs) are a class of heterogeneous materials with continuous variation of properties over their volume. Generally, these materials are composites formed by smooth gradation of two or more constituent phases along certain dimensions of a structure. This gradation can be regulated by changing the volume fraction distribution of component of material from one to another in a controlled manner [2]. As a result, the thermophysical properties of material continuously vary as a function of position along a certain direction. This allows designing a functionally graded material in order to obtain optimized friction characteristics of a brake.

In general, statements of thermal problems of friction contain partial differential equations with variable coefficients. Therefore, the application of analytical methods to their solution is difficult or even impossible. Hence, numerical methods are often used to consider such problems. An FGM disc subjected to thermal load due to frictional heating while taking into account the inertial force due to the rotation of the disc was studied by Afsar and Go [3]. A 2D finite element analysis (FEA) for a circular disc with exponential variations in thermophysical properties in the radial direction was performed. An axisymmetric FEA of a brake disc, with properties distributed according to the power-law function of radial position, was executed by Shahzamanian et al. [4,5]. It was found that the gradation index of the disc material has a crucial influence on the thermomechanical behavior of the entire braking system.

A finite element thermal contact analysis of a functionally graded disc under dry friction was performed by Hosseini and Talebi [6]. The core part of the considered disc was steel, and it gradually changed through the thickness of the disc, according to a power law, approaching pure ceramic at the outer surface. It was shown that the temperature and the corresponding thermal displacements in the FGM disc are much lower than in the conventional steel disc. Furthermore, it was established that the use of an FGM brake disc may eliminate thermal cracking and wear. In particular, functionally graded materials composed of ceramic and metal perform very well in contact problems involving friction, since they combine the advantages of both components [7]. These elements mostly have a metal core in order to maintain strength and rigidity, whereas ceramic is present on the outer surfaces to resist intensive wear and elevated temperature conditions.

Separately, the study of the phenomenon of thermoelastic instability (TEI) of brake systems with FGM should be mentioned. The solution of a 2D thermal contact problem of friction for a functionally graded cermet brake disc was obtained by means of FEA [8]. They investigated TEI caused by the coupled interaction of the mechanical and thermal loads in the sliding system. Generally, this leads to the establishment of localized high-temperature zones on the contact surface, known as hot spots, which are directly attributable to the premature failure of the friction system. This instability is often called the frictionally excited TEI and occurs in tribosystems when the sliding velocity exceeds a certain critical value. It was shown that the value of the critical velocity for a functionally graded brake disc is higher than that for a conventional homogeneous disc. This conclusion was confirmed by further research investigating TEI in an FGM strip sliding uniformly against two homogeneous semi-spaces [9,10]. Assuming an exponential variation of the thermophysical properties along the thickness of an FG strip permitted obtaining an exact solution using the analytical perturbation method. Using the same methodology, the TEI of the brake modeled as an FGM semi-infinite body sliding against a homogeneous semi-space under uniform pressure taking into account the frictional heating and thermal contact resistance was investigated by Mao et al. [11]. As a result, they determined the stability boundaries of thermoelastic instability in the considered sliding system. The effect of the arbitrarily varying thermoelastic properties of the FGM on the TEI was considered by Mao et al. [12]. To simulate the distribution of the FGM properties, a homogeneous multilayered model was employed. This approach is a replacement of the continuous FGM material with a package of homogeneous layers with constant properties. The gradient was simulated by assigning different properties values to each sublayer. It was proven that the results received for the FGM strip, divided into a sufficient number of layers, were close to the results found using the corresponding exact solutions [13]. It should be noted that this conclusion is dependent on the problem under consideration, and the differences between the obtained results may be significant in some cases [14]. This is particularly true for FGM with temperature-dependent properties. A multilayered model was used in [15] in order to establish the coupled effect of the frictional heat and the thermal contact resistance. Since the homogeneous multilayered model deals with the arbitrarily varying properties of FGM, the power-law, exponential, sinusoidal, and cosinusoidal distributions of the brake disc properties were considered. The perturbation and transfer matrix methods were used to deduce the characteristic equation of the TEI problem, to obtain the relationship between the critical sliding velocity and the critical heat flux [15]. The formulated conclusions confirm that the application of ceramic-based FGM in a brake disc, consisting of ceramics at the sliding interface and steel in the middle layer, reduces the susceptibility of braking system toward TEI [9,10,11,15]. 

However, FGMs are increasingly finding applications in braking systems, in the fabrication of not only discs, but also brake pads. Experimental investigations revealed that FGMs could successfully fulfill the demands for brake pads and improve their characteristics [1,16]. The novel functionally graded ductile iron for brake pads was investigated in a well-controlled model sliding test and a disc-brake machine in [16]. The results of the tribological tests revealed a positive effect of the functional gradient of properties on the wear of the pads and the improved stability of the friction coefficient. Govindaraju et al. [1] developed and investigated Fe-based material on brake pads with graded composition. The FG specimens were subjected to a dry sliding test for studying their tribological behavior. The results were compared with the conventional brake pad specimen. It was found that the wear resistance of the functionally gradient specimen is much greater compared to the conventional pad material [1].

We note that a more comprehensive review of the literature on thermoelastic contact problems with frictional heating for functionally graded materials was provided in our previous article [17]. This article is a continuation of the research cycle started in [17], in which the case of the uniform sliding of an FGM tribosystem was considered. The present article concerns the transient thermal problem of friction during braking, which takes into account the time-dependent specific friction power due to the exponential increase in contact pressure.

## 2. Statement to the Problem

The frictional heating in a brake disc system during a single braking process is considered. Frictional elements of the system are two identical pads, located symmetrically to the brake disc. At the initial time moment t=0, pads are pressed to the friction surfaces of the disc with uniformly distributed on the contact area and time-dependent pressure [18].

(1) $$p(t)=p_{0}p^{*}(t),\;p^{*}(t)=1-e^{-t/t_{i}},\;0\leq t\leq t_{s},$$

where $t_{i}\geq 0$ is the rise time in contact pressure from zero to the nominal value $p_{0}$, and $t_{s}$ is the time of stop. Due to the interaction of friction forces, the linear velocity of vehicle V is reduced from the initial value $V_{0}\equiv V(0)$ to zero at the stop time moment $t=t_{s}$ according to the following law [19,20]:

(2) $$V(t)=V_{0}V^{*}(t),\;V^{*}(t)=1-\frac{t}{t_{s}^{0}}+\frac{t_{i}}{t_{s}^{0}}p^{*}(t),\;t_{s}^{0}=\frac{W_{0}}{fp_{0}A_{a}V_{0}},\;0\leq t\leq t_{s},$$

where $W_{0}$ is the initial kinetic energy of the system, f is the friction coefficient, $A_{a}$ is the nominal contact area between the pad and disc, and $t_{s}^{0}$ is the braking time with constant deceleration ($t_{i}\to 0$). The braking time, taking into account the temporal profile of the velocity (Equation (2)), is determined from the stop condition $V^{*}(t_{s})=0$. For $0<t_{i}\leq 0.3t_{s}^{0}$ it was established [19] that $t_{s}≅t_{s}^{0}+0.99t_{i}$.

The sliding velocity reduction during braking is accompanied by the generation of frictional heat on the contact surface of the friction pair. In order to determine the temperature field generated in this way, the corresponding thermal problem of friction is formulated on the basis of the following assumptions:

1. The materials of the pads and the disc are functionally graded with an exponential decrease in thermal conductivity along their thickness, with invariant specific heat and density;
2. The initial temperature of all elements is the same and equal to the ambient temperature $T_{a}$;
3. The whole work of friction goes to heating the bodies, while the wear of the friction surfaces is neglected;
4. The free surfaces of the pads and the disc are adiabatic;
5. The thermal and mechanical properties and coefficient of friction are independent of the temperature T;
6. Only the change in the temperature gradient in the direction perpendicular to the friction surface is taken into account;
7. The thermal contact of friction between the pads and the disc is perfect; the temperatures of their friction surfaces during braking are the same, and the sum of the intensity of the heat fluxes directed to both elements along the normal to the contact surface is equal to the specific friction power:
  (3) $$q(t)=q_{0}q^{*}(t),\;q_{0}=fp_{0}V_{0},\;q^{*}(t)=p^{*}(t)V^{*}(t),\;0\leq t\leq t_{s},$$
  where the temporal profiles of pressure $p^{*}(t)$ and velocity $V^{*}(t)$ have the forms expressed in Equations (1) and (2), respectively;
8. Due to the symmetry with respect to the center plane of the disc, to establish the temperature of the braking system, it is sufficient to consider the contact scheme of one pad with a disc of half of its thickness.

**Figure 1:**
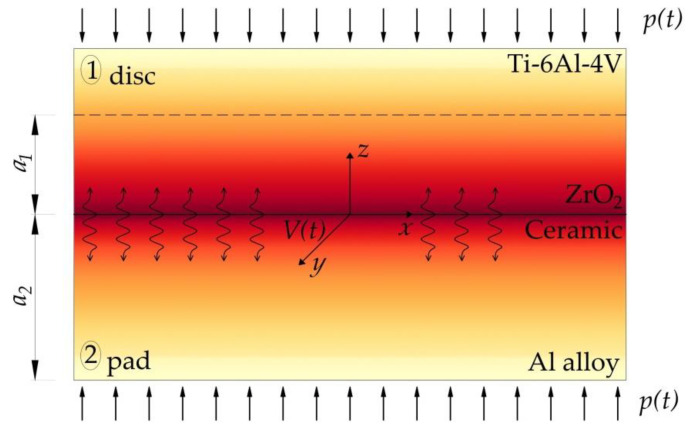
Scheme of the frictional heating in the disc–pad system.

With such assumptions, a contact scheme of two sliding semi-infinite bodies (semi-spaces) related to the Cartesian system 0xyz (Figure 1) was adopted to describe the process of frictional heating in the disc–pad system.

The temperature rise $\Theta =T-T_{a}$ was determined from solution to the following one-dimensional boundary value problem of heat conduction taking into account the generation of heat due to friction:

(4) $$\frac{\partial }{\partial z}\left[K_{1}(z)\frac{\partial \Theta (z,t)}{\partial z}\right]=c_{1}\rho_{1}\frac{\partial \Theta (z,t)}{\partial t},z>0,\;0<t\leq t_{s},$$

(5) $$\frac{\partial }{\partial z}\left[K_{2}(z)\frac{\partial \Theta (z,t)}{\partial z}\right]=c_{2}\rho_{2}\frac{\partial \Theta (z,t)}{\partial t},z<0,\;0<t\leq t_{s},$$

(6) $${K_{2}(z)\frac{\partial \Theta (z,t)}{\partial z}|}_{z=0^{-}}-{K_{1}(z)\frac{\partial \Theta (z,t)}{\partial z}|}_{z=0^{+}}=q(t),0<t\leq t_{s},$$

(7) $$\Theta (0^{-},t)=\Theta (0^{+},t),0<t\leq t_{s},$$

(8) $$\Theta (z,t)\to 0,\;\left|z\right|\to \infty ,0<t\leq t_{s},$$

(9) Θ(z,0)=0,|z|<∞,

where

(10) $$K_{l}(z)=K_{l,0}e^{\gamma_{l}\left|z\right|},\left|z\right|<\infty ,K_{l,0}\equiv K_{l}(0),\gamma_{l}\geq 0,\;l=1,\;2,$$

and function q(t) has the form expressed in Equation (3). Here and further, the subscript l indicates the parameters and quantities related to the certain element—l=1 for the disc, and l=2 for the pad. Taking into account the relations in Equation (10), the problem in Equations (6)–(9) was written in the following form:

(11) $$\frac{\partial^{2}\Theta (z,t)}{\partial z^{2}}+\gamma_{1}\frac{\partial \Theta (z,t)}{\partial z}=\frac{e^{-\gamma_{1}z}}{k_{1,0}}\frac{\partial \Theta (z,t)}{\partial t},z>0,\;0<t\leq t_{s},$$

(12) $$\frac{\partial^{2}\Theta (z,t)}{\partial z^{2}}-\gamma_{2}\frac{\partial \Theta (z,t)}{\partial z}=\frac{e^{\gamma_{2}z}}{k_{2,0}}\frac{\partial \Theta (z,t)}{\partial t},z<0,\;0<t\leq t_{s},$$

(13) $${K_{2,0}\frac{\partial \Theta (z,t)}{\partial z}|}_{z=0^{-}}-{K_{1,0}\frac{\partial \Theta (z,t)}{\partial z}|}_{z=0^{+}}=q(t),\;0<t\leq t_{s},$$

(14) $$\Theta (0^{-},t)=\Theta (0^{+},t),\;0<t\leq t_{s},$$

(15) $$\Theta (z,t)\to 0,\;\left|z\right|\to \infty ,0<t\leq t_{s},$$

(16) Θ(z,0)=0,|z|<∞,

where

(17) $$k_{l,0}=\frac{K_{l,0}}{c_{l}\rho_{l}},\;l=1,\;2$$

are the coefficients of thermal diffusivity of the materials on their contact surfaces; z=0.

## 3. Solution to the Problem

In the case of a uniform slip with a constant specific power of friction $q(t)=q_{0}$, t≥0, the solution to the problem in Equations (11)–(16) can be written in the following form [17]:

(18) $$\hat{\Theta }(z,t)=\Lambda e^{-\gamma_{1}z/2}\left[\frac{e^{-\gamma_{1}z/2}}{\left(1+\gamma_{\varepsilon }K_{\varepsilon }\right)}+\frac{4}{\gamma_{\varepsilon }}\sum_{n=1}^{\infty }\frac{\phi_{1}(z,\mu_{n})}{\Psi (\mu_{n})}e^{-p_{n}t}\right],z\geq 0,t\geq 0,$$

(19) $$\hat{\Theta }(z,t)=\Lambda e^{\gamma_{2}z/2}\left[\frac{e^{\gamma_{2}z/2}}{\left(1+\gamma_{\varepsilon }K_{\varepsilon }\right)}+\frac{4}{\gamma_{\varepsilon }}\sum_{n=1}^{\infty }\frac{\phi_{2,n}(z,)}{\Psi (\mu_{n})}e^{-p_{n}t}\right],z\leq 0,t\geq 0,$$

where

(20) $$\phi_{1}(z,\mu_{n})=J_{1}(\mu_{n})J_{1}(\gamma_{\varepsilon }\mu_{n}e^{-\gamma_{1}z/2}),\;\phi_{2}(z,\mu_{n})=J_{1}(\mu_{n})J_{1}(\gamma_{\varepsilon }\mu_{n}e^{\gamma_{2}z/2}),$$

(21) $$\Psi (\mu_{n})=\mu_{n}^{2}[(1+\gamma_{\varepsilon }K_{\varepsilon })J_{0}(\mu_{n})J_{0}(\gamma_{\varepsilon }\mu_{n})-(\gamma_{\varepsilon }+K_{\varepsilon })J_{1}(\mu_{n})J_{1}(\gamma_{\varepsilon }\mu_{n})],$$

(22) $$K_{\varepsilon }=\frac{K_{0}^{*}}{\sqrt{k_{0}^{*}}},\;\gamma_{\varepsilon }=\gamma^{*}\sqrt{k_{0}^{*}},\;K_{0}^{*}=\frac{K_{1,0}}{K_{2,0}},\;k_{0}^{*}=\frac{k_{1,0}}{k_{2,0}},\;\gamma^{*}=\frac{\gamma_{1}}{\gamma_{2}},\;\Lambda =\frac{q_{0}}{\gamma_{2}K_{2,0}},$$

(23) $$p_{n}=0.25k_{1,0}\gamma_{1}^{2}\mu_{n}^{2},$$

$\mu_{n}>0,\;n=1,2,3,\ldots$, are the real roots of the following functional equation:

(24) $$J_{0}(\gamma_{\varepsilon }\mu )J_{1}(\mu )+K_{\varepsilon }J_{0}(\mu )J_{1}(\gamma_{\varepsilon }\mu )=0,$$

where $J_{k}(x)$ denotes the Bessel functions of the first kind of the *k-*th order [21].

The temperature rise Θ(z,t) corresponding to the specific friction power q(t) in Equation (3) is searched on the basis of Duhamel’s formula [22].

(25) $$\Theta (z,t)=\frac{\partial }{\partial t}\int_{0}^{t}q^{*}(t-s)\hat{\Theta }(z,s)ds,\;0<t\leq t_{s},$$

where $\hat{\Theta }(z,t)$ is the temperature rise in Equations (18)–(24) for constant specific friction power $q(t)=q_{0}$. Taking into account the solutions to Equations (18) and (19) in Duhamel’s integral (Equation (25)), it was achieved that

(26) $$\Theta (z,t)=\Lambda e^{-\gamma_{1}z/2}\left[\frac{e^{-\gamma_{1}z/2}}{\left(1+\gamma_{\varepsilon }K_{\varepsilon }\right)}q^{*}(t)+\frac{4}{\gamma_{\varepsilon }}\sum_{n=1}^{\infty }\frac{\phi_{1}(z,\mu_{n})}{\Psi (\mu_{n})}{G^{\prime }}_{n}(t)\right],z\geq 0,0\leq t\leq t_{s},$$

(27) $$\Theta (z,t)=\Lambda e^{\gamma_{2}z/2}\left[\frac{e^{\gamma_{2}z/2}}{\left(1+\gamma_{\varepsilon }K_{\varepsilon }\right)}q^{*}(t)+\frac{4}{\gamma_{\varepsilon }}\sum_{n=1}^{\infty }\frac{\phi_{2}(z,\mu_{n})}{\Psi (\mu_{n})}{G^{\prime }}_{n}(t)\right],z\leq 0,0\leq t\leq t_{s},$$

where ${G^{\prime }}_{n}(t)$ is a derivative of the function $G_{n}(t)$, which is determined as

(28) $$G_{n}(t)=\int_{0}^{t}q^{*}(t-s)e^{-p_{n}t}dt,n=1,2,3,\ldots$$

Substituting the temporal profile of the specific power of friction $q^{*}(t)$ in Equation (3) into Equation (28) yielded the following equation:

(29) $$G_{n}(t)=G_{n,1}(t)-\frac{1}{t_{s}^{0}}G_{n,2}(t)+\frac{t_{i}}{t_{s}^{0}}G_{n,3}(t),n=1,2,3,\ldots ,$$

where

(30) $$G_{n,1}(t)=\int_{0}^{t}p^{*}(t-s)e^{-p_{n}t}dt,\;G_{n,2}(t)=\int_{0}^{t}(t-s)p^{*}(t-s)e^{-p_{n}t}dt,\;\;G_{n,3}(t)=\int_{0}^{t}[p^{*}(t-s){]}^{2}e^{-p_{n}t}dt.$$

The calculations of integrals in Equation (30) taking into account the time profile of contact pressure $p^{*}(t)$ (1), give

(31) $$G_{n,1}(t)=p_{n}^{-1}(1-e^{-p_{n}t})+a_{n}^{-1}(e^{-p_{n}t}-e^{-t/t_{i}}),$$

(32) $$G_{n,2}(t)=t(p_{n}^{-1}-a_{n}^{-1}e^{-t/t_{i}})-p_{n}^{-2}(1-e^{-p_{n}t})-a_{n}^{-2}(e^{-p_{n}t}-e^{-t/t_{i}}),$$

(33) $$G_{n,3}(t)=p_{n}^{-1}(1-e^{-p_{n}t})+2a_{n}^{-1}(e^{-p_{n}t}-e^{-t/t_{i}})-b_{n}^{-1}(e^{-p_{n}t}-e^{-2t/t_{i}}),$$

where

(34) $$a_{n}=p_{n}-t_{i}^{-1}\neq 0,\;b_{n}=p_{n}-2t_{i}^{-1}\neq 0n=1,2,3,\ldots$$

If for any n=k, k=1,2,…, the equality $p_{k}=t_{i}^{-1}$ ($a_{k}=0,\;b_{k}=-t_{i}^{-1}$) is true, then the integration of the Equation (30) gives

(35) $$G_{k,1}(t)=t_{i}(1-e^{-t/t_{i}})-te^{-t/t_{i}},$$

(36) $$G_{k,2}(t)=t_{i}[t-t_{i}(1-e^{-t/t_{i}})]-0.5t^{2}e^{-t/t_{i}},$$

(37) $$G_{k,3}(t)=t_{i}(1-e^{-2t/t_{i}})-2te^{-t/t_{i}}.$$

On the other hand, for $p_{k}=2t_{i}^{-1}$ ($a_{k}=t_{i}^{-1},\;b_{k}=0$) it was obtained that

(38) $$G_{k,1}(t)=0.5t_{i}{\left(1-e^{-t/t_{i}}\right)}^{2},$$

(39) $$G_{k,2}(t)=0.5t_{i}[t-0.5t_{i}(1-e^{-2t/t_{i}})]-t_{i}[t-t_{i}(1-e^{-t/t_{i}})]e^{-t/t_{i}},$$

(40) $$G_{k,3}(t)=0.5t_{i}(1-e^{-2t/t_{i}})-2t_{i}(1-e^{-t/t_{i}})e^{-t/t_{i}}+te^{-2t/t_{i}}.$$

Substituting the function $G_{n,i}(t)$, i=1,2,3 in Equations (31)–(33) into the right side of Equation (29) yields

(41) $$\begin{array}{ll} G_{n}(t) & =\left(1+\frac{t_{i}}{t_{s}^{0}}+\frac{1}{t_{s}^{0}p_{n}}\right)\frac{\left(1-e^{-p_{n}t}\right)}{p_{n}}-\left(1+\frac{2t_{i}}{t_{s}^{0}}+\frac{1}{t_{s}^{0}a_{n}}\right)\frac{\left(e^{-t/t_{i}}-e^{-p_{n}t}\right)}{a_{n}}+\\ & +\frac{t_{i}(e^{-2t/t_{i}}-e^{-p_{n}t})}{t_{s}^{0}b_{n}}-\frac{t}{t_{s}^{0}}\left(\frac{1}{p_{n}}-\frac{e^{-t/t_{i}}}{a_{n}}\right),\;0\leq t\leq t_{s},\;n=1,2,\ldots . \end{array}$$

The searched derivative of the function $G_{n}(t)$ in Equation (41), meeting the conditions in Equation (34), has the following form:

(42) $$\begin{array}{ll} {G^{\prime }}_{n}(t) & =\left(1+\frac{t_{i}}{t_{s}^{0}}\right)e^{-p_{n}t}-\frac{\left(1-e^{-p_{n}t}\right)}{t_{s}^{0}p_{n}}+\left(1+\frac{2t_{i}}{t_{s}^{0}}+\frac{1}{t_{s}^{0}a_{n}}\right)\frac{\left(t_{i}^{-1}e^{-t/t_{i}}-p_{n}e^{-p_{n}t}\right)}{a_{n}}+\\ & +\frac{1}{t_{s}^{0}a_{n}}\left(1-\frac{t}{t_{i}}\right)e^{-t/t_{i}}-\frac{t_{i}(2t_{i}^{-1}e^{-2t/t_{i}}-p_{n}e^{-p_{n}t})}{t_{s}^{0}b_{n}},\;0\leq t\leq t_{s},\;n=1,2,\ldots . \end{array}$$

Proceeding in a similar manner, from Equations (29) and (35)–(40), the derivative for $p_{k}=t_{i}^{-1}$ was found.

(43) $${G^{\prime }}_{k}(t)=\frac{t}{t_{s}^{0}}\left(3+\frac{t_{s}^{0}}{t_{i}}-\frac{t}{2t_{i}}\right)e^{-t/t_{i}}+\frac{t_{i}}{t_{s}^{0}}\left(2e^{-2t/t_{i}}-e^{-t/t_{i}}-1\right),$$

That for $p_{k}=2t_{i}^{-1}$ was also found.

(44) $${G^{\prime }}_{k}(t)=\left(1+4\frac{t_{i}}{t_{s}^{0}}\right)\left(e^{-t/t_{i}}-e^{-2/t_{i}}\right)-\frac{t_{i}}{2t_{s}^{0}}\left(1-e^{-2t/t_{i}}\right)-\frac{t}{t_{s}^{0}}\left(e^{-t/t_{i}}+2e^{-2t/t_{i}}\right).$$

Approaching $p_{n}\to 0$ ($a_{n}\to -t_{i}^{-1}$, $b_{n}\to -2t_{i}^{-1}$), the limit of Equation (42) was found.

(45) $$\begin{array}{ll} \underset{p_{n}\to 0}{\lim}{G^{\prime }}_{n}(t) & =1+\frac{t_{i}}{t_{s}^{0}}-\frac{t}{t_{s}^{0}}-\left(1+\frac{t_{i}}{t_{s}^{0}}\right)e^{-t/t_{i}}+\frac{t_{i}}{t_{s}^{0}}e^{-2t/t_{i}}-\frac{t_{i}}{t_{s}^{0}}\left(1-\frac{t}{t_{i}}\right)e^{-t/t_{i}}=\\ & =\left(1-e^{-t/t_{i}}\right)\left[1-\frac{t}{t_{s}^{0}}+\frac{t_{i}}{t_{s}^{0}}\left(1-e^{-t/t_{i}}\right)\right]=p^{*}(t)\left[1-\frac{t}{t_{s}^{0}}+\frac{t_{i}}{t_{s}^{0}}p^{*}(t)\right]=q^{*}(t), \end{array}\;0\leq t\leq t_{s},$$

where $p^{*}(t)$ and $q^{*}(t)$ are the dimensionless temporal profiles of pressure (Equation (1)) and specific friction power (Equation (3)), respectively, where the function $q^{*}(t)$ occurs beyond the sign of the sum in the solutions in Equations (26)–(28).

It should be noted that, at the initial time moment, from Equation (3), it follows $q^{*}(0)=0$ and, from Equation (42), taking into account Equation (34), it was found that

(46) $$\begin{array}{ll} {G^{\prime }}_{n}(0) & =1+\frac{t_{i}}{t_{s}^{0}}+\left(1+\frac{2t_{i}}{t_{s}^{0}}+\frac{1}{t_{s}^{0}a_{n}}\right)\frac{\left(t_{i}^{-1}-p_{n}\right)}{a_{n}}-\frac{t_{i}(2t_{i}^{-1}-p_{n})}{t_{s}^{0}b_{n}}+\frac{1}{t_{s}^{0}a_{n}}=\\ & =1+\frac{t_{i}}{t_{s}^{0}}-1-\frac{2t_{i}}{t_{s}^{0}}-\frac{1}{t_{s}^{0}a_{n}}+\frac{t_{i}}{t_{s}^{0}}+\frac{1}{t_{s}^{0}a_{n}}=0,\;p_{n}\neq t_{i}^{-1}\vee p_{n}\neq 2t_{i}^{-1}\;,\;n=1,2,\ldots \;. \end{array}$$

If $p_{k}=t_{i}^{-1}$ or $p_{k}=2t_{i}^{-1}$, then, from Equations (43) and (44), it follows that ${G^{\prime }}_{k}(0)=0$. In this way, it was shown that the solution in Equations (26) and (27) meets the initial condition in Equation (16). 

In the special case for $t_{i}\to 0$, when the pressure p(t) in Equation (1) attains the nominal value $p_{0}$ immediately, and the velocity V(t) in Equation (2) reduces linearly (braking with constant deceleration), the dimensionless temporal profile of the specific friction power $q^{*}(t)$ and function ${G^{\prime }}_{n}(t)$ in Equation (42) takes the following form:

(47) $$q^{*}(t)=1-\frac{t}{t_{s}^{0}},\;{G^{\prime }}_{n}(t)=e^{-p_{n}t}-\frac{\left(1-e^{-p_{n}t}\right)}{t_{s}^{0}p_{n}},\;0\leq t\leq t_{s}^{0}.$$

From Equations (47) and (48), it follows that $q^{*}(0)=1$, ${G^{\prime }}_{n}(0)=1$. This means that fulfillment of the initial condition in Equation (16) in this case is possible when the following equality is satisfied:

(48) $$\sum_{n=1}^{\infty }\frac{\phi_{1}(z,\mu_{n})}{\Psi (\mu_{n})}=\frac{0.25\gamma_{\varepsilon }}{1+\gamma_{\varepsilon }K_{\varepsilon }}e^{-\gamma_{1}z/2},z\geq 0,\;\sum_{n=1}^{\infty }\frac{\phi_{2}(z,\mu_{n})}{\Psi (\mu_{n})}=\frac{0.25\gamma_{\varepsilon }}{1+\gamma_{\varepsilon }K_{\varepsilon }}e^{\gamma_{2}z/2},z\leq 0,$$

where functions $\phi_{l}(z,\mu_{n})$, l=1,2, and $\Psi (\mu_{n})$ have the form in Equations (20)–(22). The validation of the summation of functional series in Equation (48) was performed numerically.

## 4. Dimensionless Form of Solution

The following denotes are introduced:

(49) $$\zeta =\frac{z}{a},\;\tau =\frac{k_{1,0}t}{a^{2}},\;\tau_{s}=\frac{k_{1,0}t_{s}}{a^{2}},\;\tau_{s}^{0}=\frac{k_{1,0}t_{s}^{0}}{a^{2}},\;\tau_{i}=\frac{k_{1,0}t_{i}}{a^{2}},\;\gamma_{l}=\frac{\gamma_{l}^{*}}{a},l=1,2,\;\;\Theta_{0}=\frac{q_{0}a}{K_{1,0}},\;\Theta^{*}=\frac{\Theta }{\Theta_{0}},$$

where $a=\max\{a_{1},\;a_{2}\}$, $a_{l}$, and l=1,2 is the thickness of the friction pair element, which actively participates in the absorption of heat. This is the distance from the friction surface, on which the temperature is 5% of maximum values achieved on this surface [23].

(50) $$a_{l}=\sqrt{3k_{l,0}t_{s}},\;l=1,2.$$

Taking into account the denotes in Equation (49) in Equations (1)–(3), (20), and (42), and the solutions in Equations (26) and (27), the dimensionless temperature rise can be written in the following form:

(51) $$\Theta^{*}(\zeta ,\tau )=\frac{K_{0}^{*}}{\gamma_{2}^{*}}e^{-\gamma_{1}^{*}\zeta /2}\left[\frac{e^{-\gamma_{1}^{*}\zeta /2}}{\left(1+\gamma_{\varepsilon }K_{\varepsilon }\right)}q^{*}(\tau )+\frac{4}{\gamma_{\varepsilon }}\sum_{n=1}^{\infty }\frac{\phi_{1}^{*}(\zeta ,\mu_{n})}{\Psi (\mu_{n})}G^{\prime }(\tau ,\mu_{n})\right],\;\zeta \geq 0,\;0\leq \tau \leq \tau_{s},$$

(52) $$\Theta^{*}(\zeta ,\tau )=\frac{K_{0}^{*}}{\gamma_{2}^{*}}e^{\gamma_{2}^{*}\zeta /2}\left[\frac{e^{\gamma_{2}^{*}\zeta /2}}{\left(1+\gamma_{\varepsilon }K_{\varepsilon }\right)}q^{*}(\tau )+\frac{4}{\gamma_{\varepsilon }}\sum_{n=1}^{\infty }\frac{\phi_{2}^{*}(\zeta ,\mu_{n})}{\Psi (\mu_{n})}G^{\prime }(\tau ,\mu_{n})\right],\;\zeta \leq 0,\;\;0\leq \tau \leq \tau_{s},$$

where

(53) $$q^{*}(\tau )=p^{*}(\tau )\left[1-\frac{\tau }{\tau_{s}^{0}}+\frac{\tau_{i}}{\tau_{s}^{0}}p^{*}(\tau )\right],\;p^{*}(\tau )=1-e^{-\tau /\tau_{i}},$$

(54) $$\phi_{1}^{*}(\zeta ,\mu_{n})=J_{1}(\gamma_{\varepsilon }\mu_{n})J_{1}(\mu_{n}e^{-\gamma_{1}^{*}\zeta /2}),\;\phi_{2}^{*}(\zeta ,\mu_{n})=J_{1}(\mu_{n})J_{1}(\gamma_{\varepsilon }\mu_{n}e^{\gamma_{2}^{*}\zeta /2}),$$

(55) $$\begin{array}{r} {G^{\prime }}_{n}(\tau )=\left(1+\frac{\tau_{i}}{\tau_{s}^{0}}\right)e^{-\lambda_{n}\tau }-\frac{\left(1-e^{-\lambda_{n}\tau }\right)}{\tau_{s}^{0}\lambda_{n}}+\left(1+\frac{2\tau_{i}}{\lambda_{s}^{0}}+\frac{1}{\tau_{s}^{0}\alpha_{n}}\right)\frac{\left(\tau_{i}^{-1}e^{-\tau /\tau_{i}}-\lambda_{n}e^{-\lambda_{n}\tau }\right)}{\alpha_{n}}-\\ -\frac{\tau_{i}(2\tau_{i}^{-1}e^{-2\tau /\tau_{i}}-\lambda_{n}e^{-\lambda_{n}\tau })}{\tau_{s}^{0}\beta_{n}}+\frac{1}{\tau_{s}^{0}\alpha_{n}}\left(1-\frac{\tau }{\tau_{i}}\right)e^{-\tau /\tau_{i}},\; \end{array}\;\;\alpha_{n}=\lambda_{n}-\tau_{i}^{-1}\neq 0,\;\beta_{n}=\lambda_{n}-2\tau_{i}^{-1}\neq 0,$$

(56) $$\lambda_{n}={\left(0.5\gamma_{1}^{*}\mu_{n}\right)}^{2},\;n=1,2,\ldots ,$$

(57) $$\tau_{s}≅\tau_{s}^{0}+0.99\tau_{i},\;0<\tau_{i}\leq 0.3\tau_{s}^{0}.$$

Function $\Psi (\mu_{n})$ is given by Equation (21), and numbers $\mu_{n}>0$ are the real roots of the functional Equation (24). From Equations (43) and (44), it follows that

(58) $${G^{\prime }}_{k}(\tau )=\frac{\tau }{\tau_{s}^{0}}\left(3+\frac{\tau_{s}^{0}}{\tau_{i}}-\frac{\tau }{2\tau_{i}}\right)e^{-\tau /\tau_{i}}+\frac{\tau_{i}}{\tau_{s}^{0}}\left(2e^{-2\tau /\tau_{i}}-e^{-\tau /\tau_{i}}-1\right),\;\lambda_{k}=\tau_{i}^{-1},$$

(59) $${G^{\prime }}_{k}(\tau )=\left(1+4\frac{\tau_{i}}{\tau_{s}^{0}}\right)\left(e^{-t/t_{i}}-e^{-2\tau /\tau_{i}}\right)-\frac{\tau_{i}}{2\tau_{s}^{0}}\left(1-e^{-2\tau /\tau_{i}}\right)-\frac{\tau }{\tau_{s}^{0}}\left(e^{-\tau /\tau_{i}}+2e^{-2\tau /\tau_{i}}\right),\;\lambda_{k}=2\tau_{i}^{-1}.$$

Substituting ζ=0 into Equations (51), (52), and (54), the dimensionless temperature rise on the contact surface can be written in the following form:

(60) $${G^{\prime }}_{k}(\tau )=\left(1+4\frac{\tau_{i}}{\tau_{s}^{0}}\right)\left(e^{-t/t_{i}}-e^{-2\tau /\tau_{i}}\right)-\frac{\tau_{i}}{2\tau_{s}^{0}}\left(1-e^{-2\tau /\tau_{i}}\right)-\frac{\tau }{\tau_{s}^{0}}\left(e^{-\tau /\tau_{i}}+2e^{-2\tau /\tau_{i}}\right),\;\lambda_{k}=2\tau_{i}^{-1},$$

where

(61) $$\phi^{*}(\mu_{n})\equiv \phi_{1}^{*}(0,\mu_{n})=\phi_{2}^{*}(0,\mu_{n})=J_{1}(\gamma_{\varepsilon }\mu_{n})J_{1}(\mu_{n}).$$

In case of braking with constant deceleration ($\tau_{i}\to 0$), from Equation (47), it can be obtained that

(62) $$q^{*}(\tau )=1-\frac{\tau }{\tau_{s}^{0}},\;G^{\prime }(\tau ,\mu_{n})=e^{-\lambda_{n}\;\tau }-\frac{1}{\tau_{s}^{0}\lambda_{n}}(1-e^{-\lambda_{n}\;\tau }),\;0\leq \tau \leq \tau_{s}^{0}.$$

It should be noted that the exact solution to the problem considering the contact scheme of friction for two semi-infinite bodies, made of homogeneous materials ($\gamma_{1}=\gamma_{2}=0$), with account of the time of contact pressure increase, was achieved in [19]. A special case of this solution—braking with constant deceleration—was investigated in [24].

## 5. Numerical Analysis

On the basis of the obtained exact solutions in Equations (51), (52), and (60), the calculations of the temperature generated due to friction in the disc–pad system during single braking were performed. Materials of the friction surfaces of elements were zirconium dioxide (l=1) and the other ceramic (l=2). With the distance from these surfaces deeper into the bodies, their thermal conductivity coefficients increased exponentially in accordance with Equation (10), reaching at the effective depths $a_{l}$, l=1,2 values corresponding to titanium and aluminum alloys, respectively. The thermal properties of the abovementioned materials are listed in Table 1.

Values of the remaining input parameters were as follows: $A_{a}=0.442\cdot 10^{-2}m^{2}$, f=0.27, $p_{0}=0.607\;\mathrm{MPa}$, $T_{0}=20{\;}^{\circ }C$, $V_{0}=23.8\;m\;s^{-1}$, and $W_{0}=103.54\;\mathrm{kJ}$ [26]. From Equation (2), the braking time with constant deceleration was found $t_{s}^{0}=12\;s$ and, next, the stop time $t_{s}=12.49\;s.$ This allowed determining from Equation (50) the effective depths of heat penetration $a_{1}=5.556\;\mathrm{mm}$ and $a_{2}=6.435\;\mathrm{mm}$, as well as the value of the scaling parameter $a=a_{2}$. According to the methodology, described in detail in [17], the dimensionless parameters of the material gradient were also established as $\gamma_{1}^{*}=1.28$ and $\gamma_{2}^{*}=4.05$.

**Figure 2:**
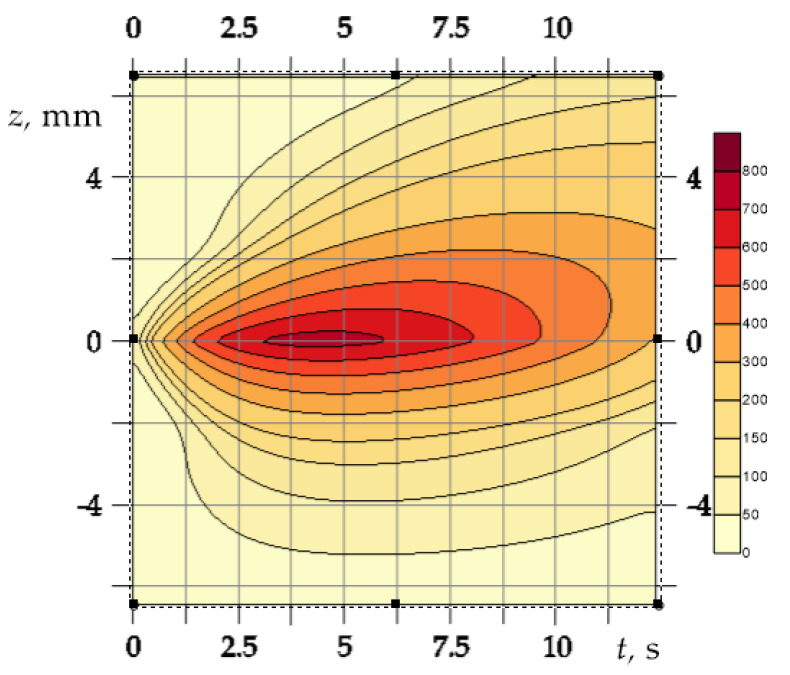
Isotherms of the temperature rise Θ(z,t) in the disc and the pad at $t_{i}=0.5\;s$.

Isotherms of the temperature rise Θ(z,t) inside the elements of the friction pair are illustrated in Figure 2. The most heated ($\Theta =800÷943{\;}^{\circ }C$) was a narrow, approximately 0.5 mm thick, near-surface area that appeared ≈3 s after start of braking. The lifetime of such a high-temperature area is ≈3 s. The friction surfaces of both elements were cooled down until the stop time moment. At the stop moment, the distance from the friction surface, where the noticeable temperature occurs in the disc was greater than in the pad.

**Figure 3:**
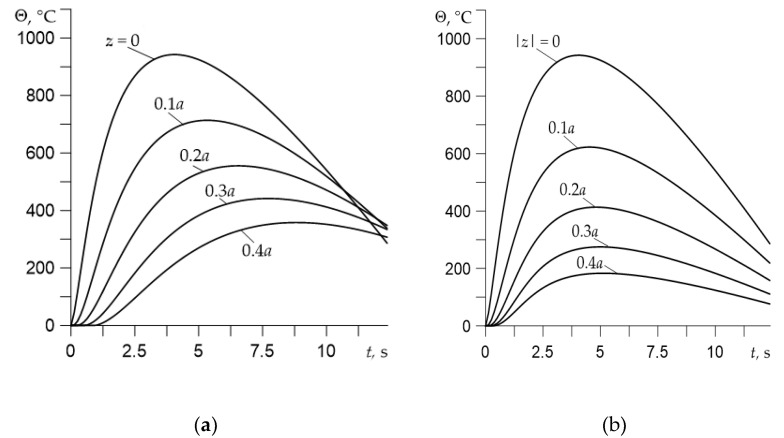
Evolutions of the temperature rise Θ(z,t) during braking at $t_{i}=0.5\;s$ for different distances from the friction surface: (**a**) the disc; (**b**) the pad.

Evolutions of the temperature rise Θ(z,t) during braking on the contact surface and inside the friction elements on different depths are presented in Figure 3. At the beginning of braking, the temperature on the friction surfaces z=0 rapidly increases over time, achieving the maximum value $\Theta_{\max}=943^{\circ }C$ at the moment $t_{\max}=5\;s$. This is followed by a period of cooling of these surfaces until it stops. The temporal profiles of the temperature inside the disc and the pad also have a similar shape. However, the known “delay” effect is visible in the disc, which is that the time to reach the maximum temperature increases with the distance from the contact surface. At the same time, this effect is almost imperceptible. Noteworthy is also the process of rapid cooling on the friction surface of disc after reaching maximum $\Theta_{\max}$; at the stop moment, the temperature inside the disc is higher than on the surface. Again, this effect does not occur in the pad material.

**Figure 4:**
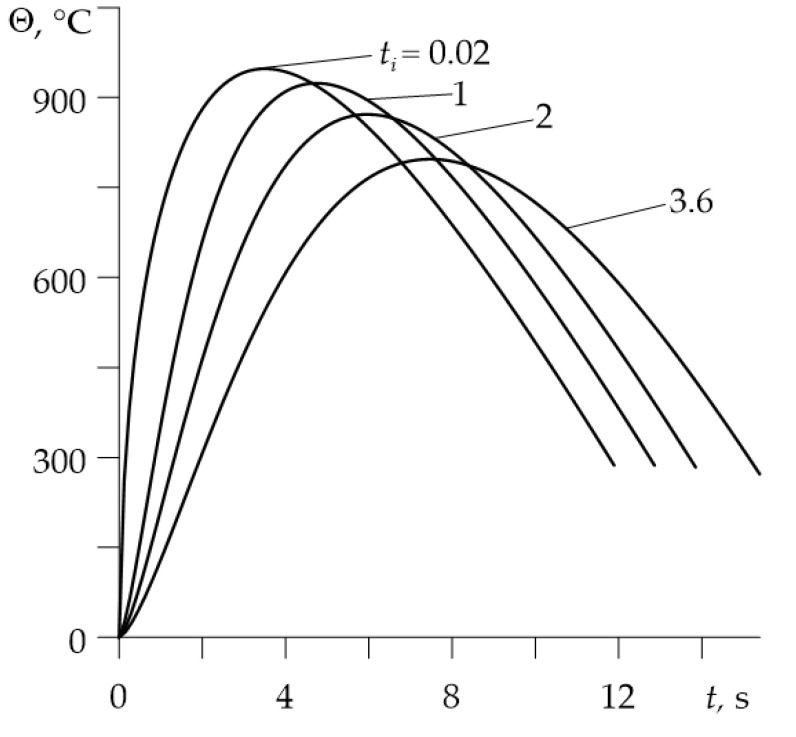
Evolutions of the temperature rise Θ(0,t) during braking for different values of the time $t_{i}$ of contact pressure increase.

**Figure 5:**
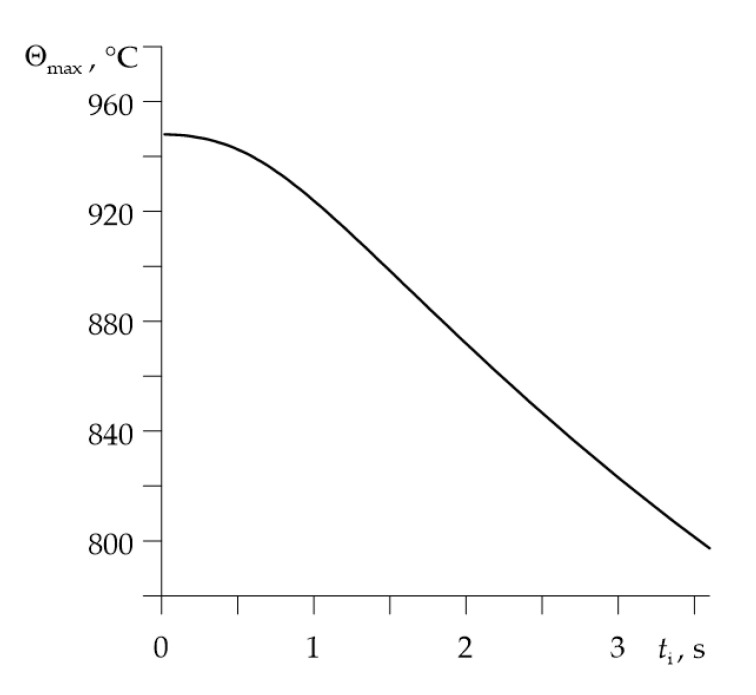
Dependence of the maximum temperature rise $\Theta_{\max}$ on the time $t_{i}$ of contact pressure increase.

Variations of the temperature during braking on the friction surfaces of disc and pad for different times of contact pressure increase are demonstrated in Figure 4. Extending the time of achieving the nominal value of pressure causes a drop of maximum temperature on the contact surface, while increasing the braking time. The effect of temperature drop with the growth of time of pressure increase is also presented in Figure 5.

**Figure 6:**
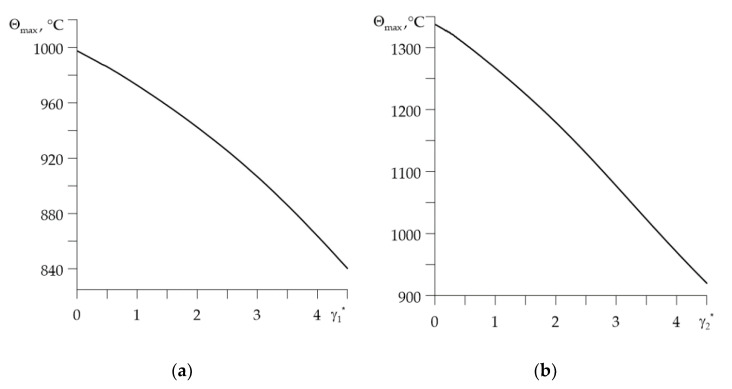
Dependence of the maximum temperature rise $\Theta_{\max}$ at $t_{i}=0.5\;s$ on the dimensionless gradient of material: (**a**) $\gamma_{1}^{*}$ for $\gamma_{2}^{*}=4.05$; (**b**) $\gamma_{2}^{*}$ for $\gamma_{1}^{*}=1.28$.

The influence of dimensionless parameters of material gradients $\gamma_{l}^{*}$, l=1,2 on the dimensionless maximum temperature $\Theta_{\max}^{*}$ on the contact surface is illustrated in Figure 6. It shows that an increase in the core material volume fraction in selected FGMs (Ti-6Al-4V for disc and aluminum alloy for pad) causes a decrease in the maximum temperature in the brake. The biggest drop in $\Theta_{\max}^{*}$ occurs when the gradient of the pad material $\gamma_{2}^{*}$ is increased (Figure 6b). However, the highest values of $\Theta_{\max}^{*}$ are reached for the friction pair in which one of the elements is entirely made of homogeneous material. These are zirconium dioxide ${\mathrm{ZrO}}_{2}$ for the disc ($\Theta_{\max}^{*}=995{\;}^{\circ }C$ at $\gamma_{1}^{*}=0$, in Figure 6a) and the other ceramic for the pad ($\Theta_{\max}^{*}=1340{\;}^{\circ }C$ at $\gamma_{2}^{*}=0$, in Figure 6b).

## 6. Conclusions

The presented results are the continuation of an investigation from a previous article of the authors [17], in which, in the dimensionless form, a comparative, qualitative analysis was performed in order to study the influence of gradient of FGMs on the temperature during frictional heating under uniform sliding. However, in this paper the mathematical model was derived to determine the temperature field in a disc–pad system during single braking. An important and unique feature of this model was its taking into account of the time-dependent pressure and velocity for friction elements, made of functionally graded materials with exponentially changing conductivity coefficients with thickness. The proposed model allows for a quick assessment of the brake temperature mode depending on the operational parameters, such as the time of contact pressure increase and the value of the gradient of the friction materials. The analysis was performed in the dimensional form. The friction surfaces of the materials were ceramic, and their cores were titanium alloys (disc) and aluminum alloys (pad). It was established that extending the time of pressure increase causes significant extending of the braking time and, thus, extending of the braking distance. The maximum temperature reached on the friction surfaces drops when the parameters of material gradients are increased. 

Application of the proposed model has some limitations, resulting from the simplifying assumptions made, especially the use of only an exponential function to describe the thermal conductivity changes in FGMs. In further research, it is planned to include in the formulation of the boundary value problem of heat conduction, as well as the thermal resistance on the contact surface of the disc and the pad (imperfect thermal contact of friction), and to adapt the obtained exact solution to determine the temperature of the brake during a repeated short-term mode of braking.

As shown in the results of the numerical analysis presented in this article, the maximum temperature achieved even with a single braking is quite high. With such a temperature, the necessary problem is to develop a model that takes into account the thermal sensitivity of the materials. Some steps toward implementing the exact solutions of linear problems for homogeneous materials to take into account their thermal sensitivity have already been made for a single [27] and a repetitive short-term [28] braking modes. On the basis of this methodology, the development of appropriate models for FGM has begun.

Another problem caused by high temperatures is a reduction in the strength of the material, especially when the temperature exceeds the melting point of the aluminum alloy. Investigations of the strength were not the subject of this article, but they should also be considered in the future.

## Figures and Tables

**Table 1:** Thermophysical properties of the FGM components [15,25].

Element Subscript	Material	$\mathrm{Thermal}\;\mathrm{Conductivity}\;K\;[{\mathrm{Wm}}^{-1}K^{-1}]$	$\mathrm{Thermal}\;\mathrm{Diffusivity}\;k\;\times \;10^{6}\;[m^{2}s^{-1}]$
l=1	ZrO_2_	2.09	0.86
	Ti-6Al-4V	7.5	3.16
l=2	ceramic	3	1.15
	aluminum alloy	173	67.16

## Data Availability

No new data were created or analyzed in this study. Data sharing is not applicable to this article.

## Nomenclature

$a_{l}$	Effective depth of heat penetration (m)
$A_{a}$	Area of the nominal contact region ($m^{2}$)
$c_{l}$	Specific heat ($J\;{\mathrm{kg}}^{-1}K^{-1}$)
f	Coefficient of friction (dimensionless)
$J_{k}(\cdot )$	Bessel functions of the first kind of the *k-*th order
$k_{l}$	Thermal diffusivity ($m^{2}s^{-1}$)
$K_{l}$	Thermal conductivity ($W\;m^{-1}K^{-1}$)
p	Contact pressure (Pa)
$p_{0}$	Nominal value of the contact pressure (Pa)
q	Specific power of friction ($W\;m^{-2}$)
$q_{0}$	Nominal value of the specific power of friction ($W\;m^{-2}$)
t	Time (s)
$t_{i}$	Time of the contact pressure increase (s)
$t_{s}^{0}$	Stop time at braking with constant deceleration (s)
$t_{s}$	Stop time (s)
T	Temperature (${}^{\circ }C$)
$T_{a}$	Initial (ambient) temperature (${}^{\circ }C$)
V	Velocity ($m\;s^{-1}$)
$V_{0}$	Initial velocity ($m\;s^{-1}$)
$W_{0}$	Initial kinetic energy of the system (J)
x, y, z	Spatial coordinates (m)
lower l	Number of the main (l=1) and frictional (l=2) elements of the friction pair
$\gamma_{l}^{}$	Parameter of material gradient ($m^{-1}$)
$\gamma_{l}^{*}$	Parameter of material gradient (dimensionless)
$\Theta_{l}$	Temperature rise (${}^{\circ }C$)
$\Theta_{l}^{*}$	Temperature rise (dimensionless)
$\Theta_{0}$	Temperature scaling factor (${}^{\circ }C$)
$\rho_{l}$	Density ($\mathrm{kg}\;m^{-3}$)
τ	Time (dimensionless)
$\tau_{i}$	Time of contact pressure increase (dimensionless)
$\tau_{s}^{0}$	Braking time at constant deceleration (dimensionless)
$\tau_{s}$	Braking time (dimensionless)
ζ	Spatial coordinate in axial direction (dimensionless)
